# Leucine and Valine Improve Pork Tenderness by Driving Myofiber Transition, Optimizing Lipid Profiles and Enhancing Antioxidant Defense

**DOI:** 10.3390/ani16152317

**Published:** 2026-07-28

**Authors:** Xingyu Wang, Dianjing Fu, Junhui Song, Hongzhi Liu, Yutong Sun, Xinbo Zhou

**Affiliations:** College of Animal Science and Technology, Northeast Agricultural University, Harbin 150030, China; s2505012075@neau.edu.cn (X.W.); dianjingfu@163.com (D.F.); songjunhui0806@outlook.com (J.S.); s240502126@neau.edu.cn (H.L.); s210501806@neau.edu.cn (Y.S.)

**Keywords:** leucine, valine, tenderness, pork, antioxidant capacity, lipid metabolism

## Abstract

Pork tenderness is a critical quality trait. Pork with divergent shear force (*n* = 10, 20 pigs in total) revealed a positive association between tenderness and branched-chain amino acid (BCAA) concentrations. Therefore, large white pigs (initial body weight 100.88 kg) were fed a control diet or diets supplemented with 1% leucine (Leu) or valine (Val) (*n* = 6, 18 pigs in total). Compared with the control, Leu or Val significantly reduced shear force, intramuscular fat, myofiber diameter, and cross-sectional area (*p* < 0.05). These interventions were associated with a potential shift in myofiber type, indicated by upregulated *MyHC I* and *IIa* and downregulated *MyHC IIb* and *IIx* (*p* < 0.05). The supplemented groups showed lower SFA content and an n-6/n-3 ratio, alongside enriched MUFA and n-3 PUFA content (*p* < 0.05). Antioxidant capacity significantly improved across muscle, liver, and serum, evidenced by elevated T-AOC, T-SOD, GSH-Px, and CAT and reduced MDA levels (*p* < 0.05). Lipid metabolism and antioxidant genes were also significantly upregulated (*p* < 0.05). Overall, Leu and Val may improve pork tenderness and nutritional value, which appear to be associated with the modulation of myofiber type, lipid metabolism, and antioxidative status, offering a potential nutritional strategy for producing high-quality pork.

## Simple Summary

Pork tenderness is a key quality trait influencing consumer preference. Certain amino acids, the building blocks of proteins, are suspected to contribute to tenderness, but their specific roles remain unclear. In this study, we first compared the amino acid profiles of pork with varying tenderness levels and found that the more tender meat contained higher concentrations of leucine and valine. We then evaluated the effects of supplementing pig diets with these two amino acids on meat quality. The results showed that pigs receiving leucine and valine produced pork with significantly improved tenderness. In addition, the supplemented pork exhibited a healthier fatty acid profile, characterized by a higher proportion of unsaturated fatty acids and greater resistance to oxidative damage, a natural process that can degrade meat freshness and nutritional value. These quality improvements were linked to changes in the expression of genes related to myofiber transition, lipid metabolism, and antioxidant defense. Our findings demonstrate that targeted amino acid supplementation provides a practical nutritional approach to producing pork that is more tender and nutritious, helping to meet the growing consumer demand for high-quality meat.

## 1. Introduction

Pork represents a globally demanded and affordable source of high-quality protein and fatty acids [1]. Key sensory attributes, such as flavor and tenderness, coupled with overall nutritional value, are critical determinants of consumer acceptance. However, modern pig production systems, which prioritize rapid growth and leanness, have been associated with documented declines in these quality parameters [2]. This trend contrasts sharply with growing consumer demand for healthier, safer, and higher-quality pork products.

Pork quality is governed by a multitude of interconnected parameters, including fat distribution and content, pH level, flavor, and odor [3]. Lipid metabolism influences the quantity and composition of fatty acids, thereby directly affecting sensory characteristics and nutritional value. Oxidation represents a significant degradation pathway, with deleterious effects on color stability, water-holding capacity, tenderness, and flavor development [4]. This underscores the pivotal roles of lipid metabolism in determining the oxidative susceptibility of fatty acids and the significance of endogenous antioxidant systems in maintaining pork quality.

Lipids not only serve as a source of essential fatty acids but also play a crucial role in flavor development, significantly contributing to key sensory attributes such as mouthfeel and juiciness. Through the process of lipolysis, these lipids act as precursors for essential flavor compounds [5]. The ultimate impact of lipids on meat flavor is modulated by intrinsic factors, including meat type, genotype, and feeding regimen [6]. Intramuscular fat (IMF), composed mainly of phospholipids and triglycerides, is particularly susceptible to degradation. Lipolysis occurs independently among different lipid classes: triglycerides and phospholipids are hydrolyzed by endogenous lipases within the muscle tissue [7]. Compared to ruminant meats, pork generally contains higher levels of polyunsaturated fatty acids (PUFAs), which are essential for human health [8]. Consequently, lipid metabolism in pork critically influences consumer purchasing behavior by dictating the meat’s nutritional value and health implications.

The expanding global market for high-quality pork underscores the urgent need to mitigate meat quality deterioration. Oxidation, as a primary non-microbial cause of quality loss, is triggered by antemortem and post-mortem stress factors. During storage and processing, the rapid depletion of endogenous antioxidants renders lipids highly susceptible to oxidative damage [3]. The resultant oxidative alterations at both the physical and molecular levels compromise essential quality indicators, including color stability, tenderness, flavor, and nutritional value. Furthermore, the formation of peroxides poses potential health risks to consumers. Research has also demonstrated that oxidation can result in steatosis and a significant reduction in product acceptability [9]. The oxidative stability of meat is crucial for counteracting oxidative damage, extending shelf-life, and maintaining nutritional integrity during storage and processing [10]. The intrinsic antioxidant defense mechanisms within meat exert a fundamental influence on the development and preservation of pork quality [11]. Consequently, it is imperative to enhance the intrinsic antioxidant capacity of meat to maintain its sensory and nutritional attributes [12]. Strengthening these intrinsic defenses targets the fundamental mechanisms responsible for lipid stability and oxidative resilience, thereby fundamentally improving meat quality.

Given growing concerns regarding the use of synthetic additives, research has increasingly focused on identifying effective bioactive compounds capable of modulating lipid metabolism and enhancing endogenous antioxidant defenses. Various dietary interventions involving bioactive compounds, such as amino acids, have been extensively evaluated. Previous studies reported supplementation with essential amino acids, including lysine, arginine, and methionine, improves pork quality by optimizing fatty acid profiles, myofiber characteristics, and antioxidant capacity [13,14,15]. Consequently, investigating the effects of leucine (Leu) and valine (Val) on meat quality has emerged as a subject of growing scientific interest.

Branched-chain amino acids (BCAAs), comprising Leu, isoleucine, and Val, cannot be synthesized *de novo* by mammals and must be acquired entirely through dietary intake. Beyond serving as fundamental building blocks for protein synthesis, BCAAs function as potent signaling molecules that regulate various biochemical pathways [16]. Our previous investigations demonstrated that the regulatory efficacy of isoleucine on lipid accumulation and metabolic profiles substantially overlap with those of Leu [17]. Consequently, the current experimental design specifically prioritized Leu and Val; this approach allowed us to circumvent functional redundancy while explicitly elucidating their distinct contributions to meat quality. This selection is further supported by our prior findings that Leu and Val significantly modulate fat accumulation [18]. Additionally, accumulating evidence highlights the pronounced role of Leu in mitigating oxidative stress [19,20], whereas Val catabolites actively regulate fatty acid uptake and lipid partitioning [21].

Despite their documented metabolic regulatory capacities, a critical knowledge gap persists regarding whether the targeted supplementation of Leu and Val can be practically applied in the swine industry to favorably modulate lipid profiles, bolster endogenous antioxidant defense systems, and ultimately mitigate pork quality deterioration. Therefore, we hypothesized that dietary supplementation with Leu and Val could improve meat quality, suggesting involvement in lipid metabolism and enhancing the oxidative status of finishing pigs. This study aimed to evaluate the effects of Leu and Val on the lipid profiles, antioxidant capacity, and overall pork quality, thereby providing a robust theoretical foundation for their application in high-quality pork production.

## 2. Materials and Methods

### 2.1. Animals and Diets

Experiment 1 was designed as an exploratory preliminary screening to evaluate the inherent phenotypic correlation between pork tenderness and endogenous amino acid profiles. Twenty healthy castrated large white pigs with an average initial body weight of 101.56 kg were electrically stunned and slaughtered via exsanguination. Following postmortem shear force evaluations, the pigs were divided into two groups: a high shear force (HSF) group and a low shear force (LSF) group. *Longissimus lumborum* (LL) muscle samples from each group were then collected to determine the amino acid composition.

For Experiment 2, eighteen healthy castrated large white pigs with an average initial body weight of 100.88 kg were allocated to three dietary treatments using a completely randomized design: Con group (basal diet supplemented with alanine to remain isonitrogenous with the experimental groups), Leu group (basal diet supplemented with 1% Leu), Val group (basal diet supplemented with Val to remain isonitrogenous with the Leu group), and 6 replicates of 1 each. There were no significant differences in the average initial body weights among the three assigned groups. The diet was formulated according to the National Research Council nutritional standard, and the metabolizable energy (ME) was verified using standard calculation models during formulation. To minimize potential confounding factors from ingredient variability, all basal diets were manufactured from a single homogenized commercial batch. The ingredient composition and the analyzed amino acid composition are shown in Table S1. All supplemental amino acids (purity ≥ 99%) were purchased from Hebei Huayang Biological Technology Co., Ltd. (Hengshui, China). The pigs were housed individually in pens at a controlled temperature of 23 ± 2 °C under standard commercial environmental conditions, with each pig serving as an experimental unit. The feeding trial lasted for 30 days, during which all animals had ad libitum access to feed and water. Daily feed intake and overall health status were monitored consistently. All animal procedures were approved by the Animal Care and Use Committee of Northeast Agricultural University (Approval No. NEAUEC20250220).

### 2.2. Carcass Traits and Sample Collection

At the end of Experiment 2, standardized pre-slaughter handling was strictly implemented, including a 12 h overnight fast. All pigs were electrically stunned and immediately exsanguinated at a commercial slaughterhouse. To prevent potential temporal or stress-related confounding effects, the slaughter order was completely randomized across the three dietary treatments. Blood samples were collected from the anterior vena cava and centrifuged at 2000× *g* for 20 min. The resulting plasma was stored at −20 °C for subsequent analysis. At 45 min postmortem, LL muscle samples were collected from the left side of the carcass between the twelfth/thirteenth ribs. Samples designated for meat quality evaluations were stored at 4 °C. Concurrently, samples designated for biochemical and molecular analyses were snap-frozen together with liver sample in liquid nitrogen and transferred to a −80 °C freezer. To preserve sample integrity and prevent enzymatic and RNA degradation, all downstream laboratory analyses were completed within 3 months of collection.

### 2.3. Meat Quality

The meat quality parameters including meat color, pH value, cooking loss, drip loss, and shear force were measured. To prevent subjective bias, the investigators performing the meat quality measurements were blinded to the treatment allocations. Furthermore, sample processing and instrumental analyses were performed in a randomized order. Following calibration with pH 4.0 and 6.86 buffer solutions (iNESA Scientific, Shanghai, China), muscle pH was measured using a pH meter (Mettler-Toledo, Shanghai, China) at 45 min and 24 h postmortem. The pH electrode was inserted approximately 2 cm into the LL muscle. Between measurements, the electrode was rinsed with distilled water. Muscle color parameters, including lightness (L*), redness (a*), and yellowness (b*), were measured, respectively, at 45 min and 24 h post-mortem using a portable chromameter (Konica Minolta, Tokyo, Japan). Prior to measurement, the samples were allowed to bloom in ambient air for 30 min. The instrument was calibrated against a standardized white tile before use. After removing the epimysium, a cubic LL sample (approximately 3 × 3 × 3 cm) was excised, weighed, and suspended on a wire hook inside a sealed container. After 24 h of storage at 4 °C, the samples were removed from the container and reweighed. Drip loss was calculated as the percentage of weight lost. For cooking loss determination, a separate muscle sample was weighed and heated in a water bath at 80 °C for 20 min. The sample was then cooled to room temperature, surface-dried, and reweighed. The percentage change in weight was then calculated to determine cooking loss. Shear force was measured using a tenderness meter (C-LM3, Northeast Agricultural University, Harbin, China), with the crosshead speed of the probe set at 200 mm/min.

### 2.4. Amino Acid Composition

Samples were retrieved from a −80 °C freezer and homogenized. A 2 mL aliquot of each homogenate was mixed with an equal volume of 10% sulfosalicylic acid by vortexing. The mixture was incubated at 4 °C for 1 h. Following incubation, the mixtures were centrifuged at 10,000× *g* for 15 min at 4 °C, and the supernatant was collected. Each supernatant was filtered through a 0.22 µm membrane filter. Finally, the resulting filtrate was analyzed using an automatic amino acid analyzer.

### 2.5. Chemical Composition and Enzyme Activities

The IMF content was quantitatively determined using the classic Soxhlet extraction method, with petroleum ether as the solvent, and was expressed as a percentage of the wet muscle tissue. Inosine monophosphate (IMP) content was measured using an ELISA kit (JONLN, JL46778, 25–500 ng/L, Shanghai, China). The serum, liver, and LL muscle sample content of total antioxidant capacity (T-AOC, Nanjing Jiancheng Bioengineering Institute, A015-1-2, 0.2–55.2 U/mL, Nanjing, China), total superoxide dismutase (T-SOD, Nanjing Jiancheng Bioengineering Institute, A001-3-2, 0.5–122.1 U/mL, Nanjing, China), glutathione peroxidase (GSH-Px, Nanjing Jiancheng Bioengineering Institute, A005-1-2, 20–330 U, Nanjing, China), catalase (CAT, Nanjing Jiancheng Bioengineering Institute, A007-1-1, 0.2–24.8 U/mL, Nanjing, China), and malondialdehyde (MDA, Nanjing Jiancheng Bioengineering Institute, A003-1-2, 0.5–113.0 nmoL/mL, Nanjing, China) were determined using commercial kits.

### 2.6. Histological Examination

The LL muscle samples were collected immediately from the left side of the carcass and fixed overnight in 10% neutral buffered formalin. The samples were then dehydrated and embedded in paraffin wax. Sections were cut at a thickness of 4 μm, stained with hematoxylin and eosin (Sangon, Shanghai, China), and mounted. Images were acquired using a microscope equipped with a camera system (Olympus, Tokyo, Japan). All histological processing and subsequent analyses were conducted by investigators blinded to the experimental group assignments. The mean cross-sectional area (CSA) and diameter of myofibers were measured using the ImageJ 1.8.0 system.

### 2.7. Fatty Acid Composition

The fatty acids in the LL muscle were analyzed. Fresh LL muscle samples were sliced and loaded into a freeze dryer (Labconco Corp., Kansas City, MO, USA) at −50 °C for 48 h. The dried samples were ground into a fine powder and stored for subsequent fatty acid profiling. Total lipids were extracted using a mixture of chloroform and methanol (*v*:*v*, 2:1), and the solvent was subsequently evaporated under a stream of nitrogen gas. Then, 2 mL of 0.5 mol/L KOH-methanol was added to the lipid extract, and the mixture was incubated in a water bath at 95 °C for 10 min. For esterification, a 10% solution of BF3 in methanol was added, and the mixture was incubated in a shaking water bath at 80 °C for 20 min. The mixture was shaken during saponification and esterification. After cooling, 1 mL of n-hexane and 5 mL of saturated NaCl solution were added to the mixture, which was then centrifuged at 1500× *g* for 15 min. The obtained fatty acid methyl esters were finally analyzed via gas chromatograph (Shimadzu Co., Kyoto, Japan), with reference to Supelco TM FAME standards (Sigma, Ronkonkoma, NY, USA). Gas chromatography conditions were set according to previously described protocols [2].

### 2.8. Real-Time Quantitative PCR

Total RNA was isolated from the LL muscle using Trizol reagent (Thermo Fisher Scientific, Waltham, MA, USA), and cDNA was synthesized using a PrimeScript RT Reagent Kit with gDNA Eraser (TaKaRa, Dalian, China). These procedures were performed according to the manufacturers’ instructions. Real-time quantitative PCR was performed using SYBR^®^ Premix Ex Taq^TM^ II (TaKaRa, China) on an ABI PRISM 7500 SDS thermal cycler (Applied Biosystems, Carlsbad, CA, USA). Prior to the main experiments, the amplification efficiencies of all specific primer pairs were experimentally validated utilizing standard curves generated from serial dilutions of pooled cDNA. Furthermore, to validate the amplification specificity, a continuous melting curve analysis was performed at the end of the amplification protocol. A single, distinct melting peak was confirmed for all amplicons, indicating the absence of non-specific products or primer-dimers. Relative gene expression was calculated using the 2^−ΔΔCt^ method. *β-actin* was employed as the reference gene, as its cycle threshold (*Ct*) values remained stable and unaffected by the dietary treatments. The primer sequences are listed in Table S2.

### 2.9. Western Blotting

LL muscle samples (*n* = 3 independent biological replicates randomly selected per treatment group) were homogenized in a RIPA buffer supplemented with PMSF (Beyotime Biotechnology, Shanghai, China). Following centrifugation, the supernatant containing total protein was collected. Equal amounts of protein were separated via sodium dodecyl sulfate–polyacrylamide gel electrophoresis and subsequently transferred to PVDF membrane (Beyotime Biotechnology, Shanghai, China). After blocking for 2 h, the membrane was washed three times and incubated with primary antibody at 4 °C overnight. The primary antibodies included sirtuin 1 (SIRT1, Abcam, Cambridge, UK), nuclear factor erythroid 2-related factor 2 (NRF2, Wanleibio, Shenyang, China), and β-actin (Beyotime Biotechnology, China). β-actin served as the specific internal loading control for normalization. The membrane was then incubated with secondary antibody conjugated to horseradish peroxidase (Beyotime Biotechnology, China). Protein bands were visualized using an enhanced chemiluminescence (ECL) reagent and captured by Advanced Q9 Alliance system (Uvitec, Cambridge, UK) under optimized auto-exposure conditions to preserve background signals and avoid pixel saturation. The band densities were quantified using the ImageJ system. The relative target protein expression was calculated by normalizing the densitometric value of the target protein to its corresponding β-actin density.

### 2.10. Statistical Analysis

Statistical analyses were performed using SPSS 21.0 software. The normality of data distribution and the homogeneity of variance were assessed using the Shapiro–Wilk test and Levene’s test, respectively. For Experiment 1, differences were analyzed using an independent samples *t*-test. For Experiment 2, a one-way analysis of variance (ANOVA) was performed, followed by Tukey’s post-hoc test. For correlation analyses, Spearman’s rank correlation coefficients were calculated. The data are expressed as means ± standard error of the mean (SEM), and *p* < 0.05 was considered statistically significant.

## 3. Results

### 3.1. Shear Force and Amino Acid Profiles in Pork

**Figure 1:**
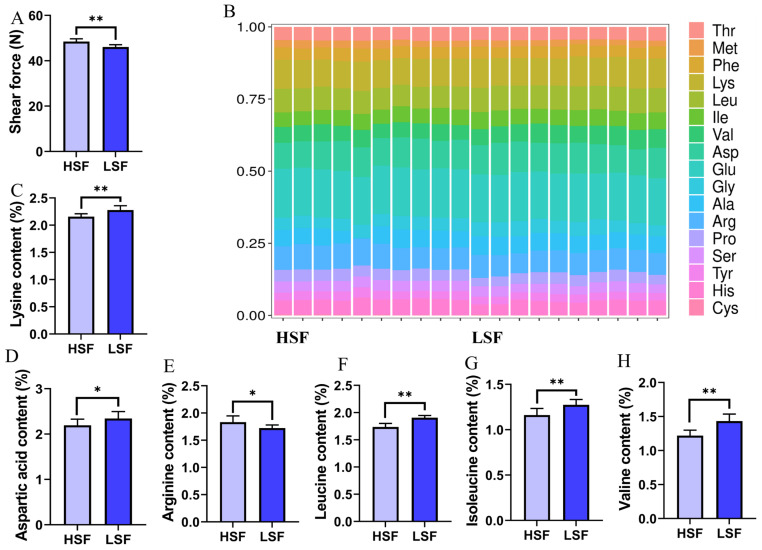
The relationship between shear force and amino acid concentration in pork. (**A**) Shear force of pork. (**B**) Amino acid contents of pork. (**C**) Lysine content of pork. (**D**) Aspartic acid content of pork. (**E**) Arginine content of pork. (**F**) Leucine content of pork. (**G**) Isoleucine content of pork. (**H**) Valine content of pork. Data are presented as mean ± SEM (*n* = 10). * means *p* < 0.05, ** means *p* < 0.01. HSF, high shear force group; LSF, low shear force group.

Lower shear force values are indicative of greater tenderness. The shear force in the HSF group was significantly higher than that in the LSF group (Figure 1A) (*p* < 0.001). Amino acid profiles also differed significantly between the two groups, with the LSF group exhibiting elevated levels of most amino acids (Figure 1B). Specifically, the concentrations of lysine, aspartic acid, and BCAAs (Leu, isoleucine, and Val) were higher in the LSF group (Figure 1C–H) (*p* = 0.001, *p* = 0.034, *p* = 0.014, *p* < 0.001, *p* = 0.001 and *p* < 0.001, respectively).

### 3.2. Effects of Leu and Val on Meat Quality

As presented in Table 1, there were no significant differences in pH, L*, a*, b*, drip loss, and cook loss between the control and amino-acid-supplemented groups. However, Leu and Val supplementation resulted in a significant decrease in shear force and IMF compared to the Con group (*p* < 0.001 and *p* = 0.009).

### 3.3. Effects of Leu and Val on Myofiber Characteristics

**Figure 2:**
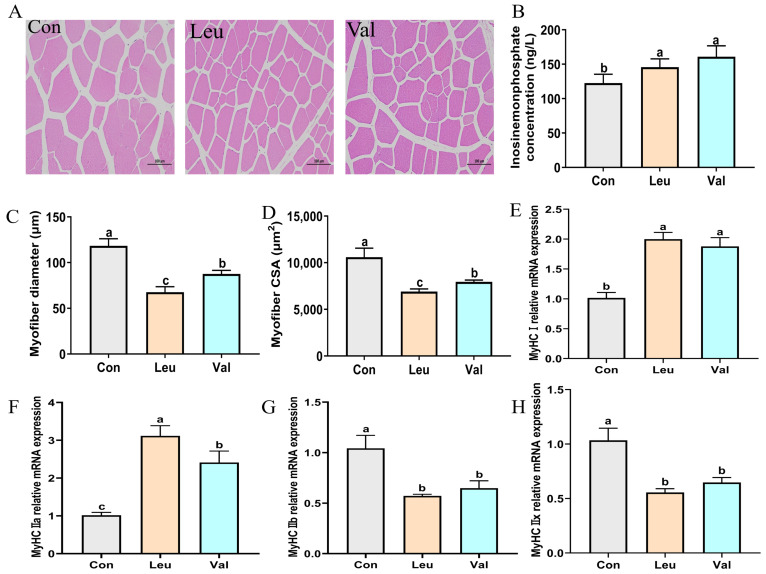
Effect of Leu and Val supplementation on myofiber characteristics. Finishing pigs were fed either a basal diet or basal diets supplemented with Leu or Val for 30 days. (**A**) HE analyses of muscle (200×), scale bar = 100 μm. (**B**) Inosine monophosphate concentration in muscle. (**C**) Myofiber diameter of each group. (**D**) Myofiber CSA of each group. (**E**) mRNA expression of *MyHC I.* (**F**) mRNA expression of *MyHC IIa*. (**G**) mRNA expression of *MyHC IIb*. (**H**) mRNA expression of *MyHC IIx*. Data are presented as mean ± SEM (*n* = 6). Mean values with different letters were considered significantly different at *p* < 0.05. CSA: Cross sectional area; *MyHC I*, Myosin heavy chain *I*; *MyHC IIa*, Myosin heavy chain *IIa*; *MyHC IIb*, Myosin heavy chain IIb; *MyHC IIx*, Myosin heavy chain *IIx*.

Myofiber characteristics were evaluated through HE staining (Figure 2A). Both Leu and Val significantly elevated the levels of IMP (*p* = 0.001), a crucial flavor compound (Figure 2B). Leu and Val supplementation resulted in a significant decrease in myofiber diameter and myofiber CSA compared with the Con group (Figure 2C,D) (*p* < 0.001). Myofiber type composition is closely associated with meat quality. Our results showed that Leu and Val significantly upregulated the expression of Myosin heavy chain (*MyHC*) *I* and *MyHC IIa* (*p* < 0.001 and *p* = 0.002) (Figure 2E,F) while significantly downregulating the expression of *MyHC IIb* and *MyHC IIx* (Figure 2G,H) (*p* = 0.003 and *p* = 0.001).

### 3.4. Effects of Leu and Val on Lipid Metabolism and Fatty Acid Composition

**Figure 3:**
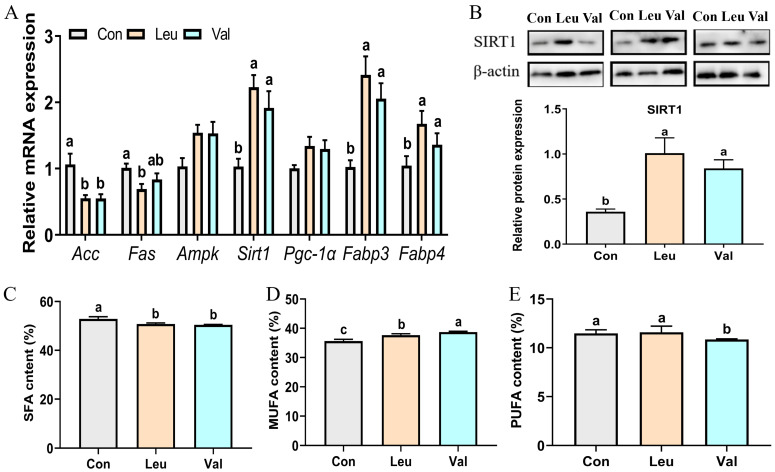
Effect of Leu and Val supplementation on lipid metabolism in the meat of finishing pigs. Finishing pigs were fed either a basal diet or basal diets supplemented with Leu or Val for 30 days. (**A**) Genes related to lipid metabolism. (**B**) Protein expression of SIRT1. (**C**) SFA content of meat. (**D**) MUFA content of meat. (**E**) PUFA content of meat. Data are presented as mean ± SEM (*n* = 6). Mean values with different letters were considered significantly different at *p* < 0.05. *Acc*, acetyl CoA carboxylase; *Fas*, fatty acid synthase; *Ampk*, adenosine monophosphate-activated protein kinase; *Sirt1*, sirtuin 1; *Pgc-1α*, peroxisome proliferator activated receptor-γ coactivator-1 alpha; *Fabp*, fatty acid-binding protein; SFA, saturated fatty acid; MUFA, mono-unsaturated fatty acids; PUFA, poly-unsaturated fatty acids.

The present study observed increased mRNA expression of *Sirt1*, fatty acid-binding protein *3* (*Fabp3*), and fatty acid-binding protein 4 (*Fabp4*) in the Leu and Val groups (*p* = 0.001, *p* = 0.001 and *p* = 0.043, respectively). In addition, decreased mRNA levels of acetyl CoA carboxylase (*Acc*) and fatty acid synthase (*Fas*) were observed (*p* = 0.004 and *p* = 0.041). However, the levels of adenosine monophosphate-activated protein kinase (*Ampk*) and peroxisome proliferator activated receptor-γ coactivator-1 alpha (*Pgc-1α*) remained unaltered (Figure 3A). An elevated level of SIRT1 protein was observed in the meat when Leu and Val were supplemented in comparison to the control group (Figure 3B) (*p* = 0.017). The content of saturated fatty acids (SFAs) was significantly decreased by Leu and Val supplementation (Figure 3C) (*p* < 0.001). Leu and Val both significantly increased the proportion of monounsaturated fatty acids (MUFAs) (*p* < 0.001). In addition, Val significantly decreased the content of PUFAs (Figure 3D,E) (*p* = 0.018).

Table 2 presents the fatty acid composition of LL muscle. Compared with the Con group, both Leu and Val supplementation significantly reduced the proportions of key saturated fatty acids (such as C14:0 and C18:0) and increased the proportion of the monounsaturated fatty acid C18:1 (*p* < 0.001). Leu supplementation significantly increased the proportions of C20:3 n3 and C20:5 n3 (*p* < 0.001 and *p* = 0.008), accompanied by a decline in C20:3 n6 (*p* < 0.001). Furthermore, the incorporation of both Leu and Val resulted in an increased content of C22:6 n3 (*p* < 0.001). The total of n-3 PUFA content was significantly increased by both Leu and Val supplementation (*p* = 0.017), while the total of n-6 PUFA was decreased by Val supplementation (*p* = 0.026). Furthermore, the supplementation of both Leu and Val led to a decrease in the n-6/n-3 ratio (*p* < 0.001).

### 3.5. Effects of Leu and Val on Antioxidant Capacity

As demonstrated in Table 3, supplementation with Leu and Val significantly increased T-SOD activity (*p* < 0.001), while Leu supplementation alone also elevated GSH-Px in serum (*p* = 0.026). In the liver, both treatments significantly decreased MDA levels (*p* < 0.001) and increased T-SOD and GSH-Px activities (*p* = 0.001 and *p* < 0.001). Additionally, Leu exhibited a notable increase in T-AOC activity in the liver (*p* = 0.03). For the Leu and Val supplementation groups, a significant decrease in LL MDA content was observed in comparison with the control group (*p* < 0.001). Leu and Val supplementation significantly increased T-AOC and T-SOD activities (*p* < 0.001). Furthermore, Leu enhanced GSH-Px and CAT activities (*p* < 0.001 and *p* = 0.01), whereas Val supplementation resulted in greater GSH-Px activity (*p* < 0.001).

### 3.6. Effects of Leu and Val on the Relationship Between Antioxidant Capacity and Meat Quality

**Figure 4:**
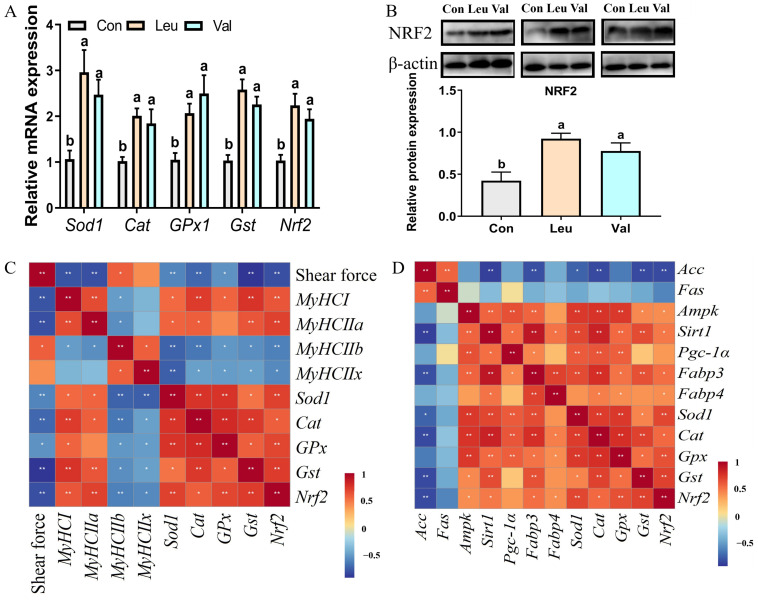
Effect of Leu and Val supplementation on levels of antioxidant-related genes in meat of finishing pigs. Finishing pigs were fed either a basal diet or basal diets supplemented with Leu or Val for 30 days. (**A**) Gene expression related to antioxidants in muscle. (**B**) NRF2 protein expression. (**C**) Correlation analysis among shear force, myosin heavy chain, and antioxidant-related genes in meat of finishing pigs. (**D**) Correlation analysis between lipid metabolism genes and antioxidant-related genes in meat of finishing pigs. Data are presented as mean ± SEM (*n* = 6). Mean values with different letters were considered significantly different at *p* < 0.05. * means *p* < 0.05; ** means *p* < 0.01.

The comparison with the control group revealed that Leu and Val significantly elevated the mRNA expression levels of *Sod1*, *Cat*, *GPx*, *Gst*, and *Nrf2* (Figure 4A) (*p* = 0.005, *p* = 0.009, *p* = 0.006, *p* < 0.001 and *p* = 0.006, respectively). Both Leu and Val supplementation significantly upregulated NRF2 protein level (*p* = 0.001), which was consistent with the mRNA expression levels (Figure 4B).

To further investigate the potential relationships between differentially expressed antioxidant capacity and meat-quality-related parameters, we conducted a correlation analysis. The results demonstrated a significant positive correlation between antioxidant genes and shear force, as well as the expression of *MyHC I* and *MyHC IIa*. *MyHC IIb* and *MyHC IIx* demonstrated a positive correlation with antioxidant genes. A significant positive correlation was identified between the expression of lipolysis genes and antioxidant-capacity-related genes; meanwhile, lipogenic genes markedly negatively correlated with MDA levels (Figure 4C,D and Figures S1 and S2). However, it is critically important to note that these correlation analyses demonstrate statistical associations between phenotypic traits and gene expressions, and they should not be interpreted as definitive evidence of biological causality or direct mechanistic pathways.

## 4. Discussion

The present study translates our exploratory phenotype-driven discovery, which identified the abundance of endogenous BCAAs as being closely associated with superior pork tenderness into a potential nutritional strategy. Among essential amino acids, the requirement for Leu is the highest in livestock due to its critical role in metabolic regulation. Our findings demonstrate that supplementation with Leu and Val significantly decreased the shear force of the LL. This tenderization, coupled with an improved lipid profile and enhanced oxidative stability, underscores the bio-efficacy of Leu and Val in producing high-quality pork.

With improving living standards, an increasing number of consumers are opting for healthier pork [22]. Consequently, nutritional intervention strategies have garnered considerable attention as safe and effective approaches to improve pork quality. Shear force is a highly reliable mechanical indicator of meat tenderness.

In this study, the dietary supplementation strategy was meticulously designed to ensure both physiological relevance and experimental rigor. A supplementation dose of 1% Leu was selected based on previous swine model studies, which demonstrated that this concentration exerts positive effects without inducing amino acid toxicity, antagonism, or feed intake suppression [23]. Crucially, to isolate the specific regulatory effects of BCAAs from a generalized response to increased dietary nitrogen, the Val dose was precisely calculated to provide a nitrogen content equivalent to that of the 1% Leu treatment. Similarly, alanine was added to the Con group to maintain a strict isonitrogenous balance across all experimental groups [13,23]. These short-term doses have been confirmed to fall well within the safe physiological tolerance limits for finishing pigs. The observed reduction in the shear force of the LL muscle induced by Leu and Val supplementation is closely associated with the structural and metabolic remodeling of myofibers. Muscle fiber characteristics, specifically CSA and myofiber type composition, largely determine the biophysical properties of meat [24,25]. Our histological analyses revealed a significant reduction in both myofiber diameter and CSA following BCAA intervention, which directly contributed to the decreased shear force.

Crucially, a pivotal factor correlating with this structural miniaturization is that the BCAAs induced fast to slow myofiber type transition, which is often overlooked in lipid-focused discussions. Muscle fibers are highly plastic: meat from muscles dominated by fast-twitch glycolytic fibers (*MyHC IIx* and *IIb*) is generally tougher, whereas a higher proportion of slow-twitch oxidative fibers (*MyHC I* and *IIa*) confers a finer texture, smaller fiber diameters, and superior postmortem tenderization [26]. Our results demonstrated a significant upregulation of *MyHC I* and *IIa,* and a concomitant downregulation of *MyHC IIx* and *IIb* in the LL muscle. These structural and metabolic shifts are consistent with recent findings in swine nutrition. For instance, recent studies have demonstrated that optimizing the dietary balance of BCAAs significantly modulates muscle fiber-type transitions [27]. Furthermore, emerging microbiome and metabolome research indicates that BCAA metabolism was associated with lipid metabolism and meat quality traits [28]. Consequently, our study further emphasizes the regulatory roles of BCAAs in meat quality.

Traditionally, meat tenderness and juiciness are positively correlated with IMF content [29], which contrasts with our current findings. This observation warrants multifaceted consideration. From a biophysical perspective, our data suggest that structural miniaturization (significant reductions in myofiber diameter and CSA) and the dynamic transition towards a higher proportion of slow-twitch oxidative fibers (*MyHC I* and *IIa*) exerted a dominant physical tenderizing effect. This fundamental microstructural remodeling of the muscle tissue architecture offset the potential macroscopic toughening effects that are typically associated with the loss of IMF. Furthermore, this paradox highlights a critical practical consideration for targeted nutritional interventions. Given the traditional association between tenderness and IMF, this particular BCAA supplementation strategy may be most suitable for pig genetic lines that naturally possess relatively high inherent IMF levels, such as large white or certain indigenous fat-type breeds. In such genetic lines, a slight metabolic reduction in IMF would not compromise tenderness, while the robust structural tenderizing benefits would maximize the enhancement of overall meat quality.

The observed reduction in IMF is potentially associated with the BCAA-induced upregulation of lipid catabolic networks. Consistent with previous reports, Leu and Val may potentially reduce lipid accumulation by upregulating the expression of components within the Sirt1/LKB1 signaling axis, which modulates the NAD/NADH ratio and suppresses lipogenic transcription factors (*Acc* and *Fas*) [30]. Concurrently, FATPs are critical genes responsible for fatty acid synthesis and uptake [31]. FABP3 and FABP4 regulate the transportation of long-chain fatty acids in muscle; therefore, their upregulated expression suggests an accelerated intracellular mobilization and metabolic turnover of long-chain fatty acids [32].

The proportional composition of fatty acids in meat serves as a key indicator of nutritional value and influences tenderness [33]. Our findings demonstrated that Leu and Val optimized the fatty acid profile by significantly increasing the proportion of beneficial unsaturated fatty acids while decreasing specific SFAs. The degree of fatty acid saturation influences fat firmness, directly affecting nutritional quality and consumer acceptance. Whereas SFAs are associated with adverse health effects, PUFAs demonstrate beneficial metabolic impacts [34]. Consequently, the SFA content in the LL muscle was significantly reduced by Leu and Val supplementation.

Notably, while n-3 PUFAs are structurally susceptible to lipid peroxidation, their dietary enrichment acts as a physiological signal that stimulates the upregulation of the endogenous cellular antioxidant defense system, ultimately enhancing the overall oxidative stability and nutritional value of the meat [35]. Compared to the control group, supplementation with either amino acid markedly increased the n-3 PUFA content. Optimizing the n-6/n-3 ratio in pork contributes to better human health outcomes, as lower ratios have been shown to mitigate fat accumulation [36]. In our study, Leu and Val significantly decreased the n-6/n-3 PUFA ratio in LL muscle. Specifically, while both amino acids increased n-3 PUFA levels, Val additionally decreased n-6 PUFA content. These alterations in fatty acid profiles indicate that Leu and Val interventions effectively enhanced the nutritional value of pork. Given that fatty acids represent key lipid components modified by amino acid supplementation, the precise mechanisms suggesting involvement in lipid metabolism require further investigation. Furthermore, to compensate for potential flavor loss resulting from reduced IMF, the concurrent elevation of IMP, a critical core nucleotide responsible for umami taste, ensures that the sensory flavor characteristics are substantially maintained and enhanced [37].

Antioxidant capacity is recognized as a critical determinant of meat quality attributes [38]. The endogenous antioxidant system constitutes the primary defense against oxidative damage, wherein disruption of the oxidant–antioxidant equilibrium induces oxidative stress with detrimental consequences for pork quality [39]. Consequently, the reinforcement of antioxidant defenses is considered a strategic approach for quality enhancement. The enzymatic antioxidant framework comprises three principal components: GSH-Px, CAT, and SOD, which collectively scavenge ROS. Elevated activities of SOD and GSH-Px are associated with reduced MDA accumulation and improved tenderness [40]. Complementary biomarkers, including T-AOC and MDA concentrations, serve as reliable indicators of meat quality status. T-AOC provides an integrated assessment of antioxidant potential, while MDA, as a terminal lipid peroxidation product, quantifies the magnitude of oxidative damage [41]. Crucially, the oxidative degradation of lipids generates MDA, which, alongside protein oxidation, compromise both nutritional integrity and sensory characteristics.

In the current investigation, Leu and Val supplementation significantly enhanced the activities of GSH-Px, T-SOD, and CAT in LL muscle. A concurrent elevation of T-AOC and a reduction of MDA content were observed, indicating attenuation of oxidative stress through modulation of antioxidant pathways. Given that enzymatic activity is transcriptionally regulated, corresponding gene expression analyses reveal that Leu and Val upregulate mRNA abundance of *Cat*, *Sod1*, and *GPx*. Our data indicate that Leu and Val successfully triggered a systemic (serum and liver) and local (muscle) antioxidant response. Because NRF2 serves as the master transcriptional coordinator of cellular redox homeostasis [42], its significant upregulation observed upon BCAA supplementation suggests a potential involvement in a cytoprotective cascade, which corresponded to the elevated enzymatic activities of T-SOD, CAT, GSH-Px, and improved T-AOC. The corresponding systemic and local decline in MDA levels strongly suggests that this NRF2-associated antioxidant reinforcement creates a critical protective microenvironment [43,44,45]. This biological shield safeguards the fatty acids from peroxidation and prevents deleterious protein oxidation, thereby preserving the structural integrity, tenderness, and extended flavor stability of the high-quality pork.

From a practical perspective, the economic implications of dietary BCAA supplementation must be critically evaluated. In the current study, specific inclusion levels of Leu and Val were utilized to clearly elucidate their physiological effects on meat quality. Although the application of amino acids would increase commercial feed costs, this nutritional strategy holds substantial potential for the high-quality pork market, where consumers are willing to pay a premium price for meat with superior tenderness and healthier fatty acid profiles, thereby achieving a balance between meat quality improvement and economic viability.

Despite the promising findings, several limitations of the present study should be acknowledged. First, Experiment 1 (*n* = 10) was an exploratory screening; therefore, Leu and Val should be strictly viewed as candidate associations rather than definitive predictive biomarkers. Second, in Experiment 2, while the sample size (*n* = 6) is well-supported by literature for mechanistic molecular and histological assays, it provides limited statistical power for macroscopic animal performance and carcass evaluations. Third, as previously discussed, our mechanistic insights regarding the SIRT1 and NRF2 pathways rely primarily on expression data, necessitating future functional validations. Finally, we evaluated one supplementation dose; further dose-response trials are necessary to determine the optimal application and economic viability of this nutritional strategy. Furthermore, a formal False Discovery Rate (FDR) correction was not systematically applied across all targeted biochemical and gene expression variables to prevent an excessive inflation of Type II errors (false-negatives) in this exploratory small cohort. However, we acknowledge that the relatively large number of measured variables in this study may increase the risk of Type I error, despite the use of targeted analyses.

## 5. Conclusions

**Figure 5:**
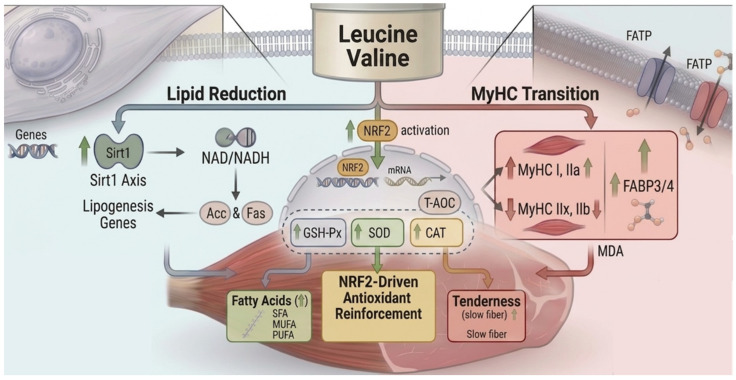
A hypothetical working model based on current findings illustrating the potential mechanisms underlying Leu- and Val-mediated improvement of pork quality. Promotion is indicated by green upward arrows; Inhibition is denoted by red downward arrows.

In summary, the present study investigated the effects of dietary supplementation with 1% Leu or Val on the meat quality of finishing pigs over a 30-day period (Figure 5). Our results demonstrated that both Leu and Val supplementation significantly reduced meat shear force, thereby improving tenderness. These improvements were accompanied by a structural shift towards slow-twitch oxidative myofibers and a reduction in myofiber diameter. Furthermore, the supplementation optimized the intramuscular fatty acid profile by enriching beneficial n-3 PUFAs and MUFAs while decreasing the n-6/n-3 ratio and SFA content. Additionally, the treatments enhanced both systemic and local antioxidant capacities, effectively mitigating lipid peroxidation. While our gene and protein expression data suggest that these beneficial changes are associated with the upregulation of lipid catabolism and the NRF2 antioxidant pathway, further functional studies are required to conclusively establish the underlying molecular mechanisms. Overall, targeted dietary inclusion of Leu or Val represents a practical nutritional strategy for producing high-quality, tender, and oxidatively stable pork.

## Figures and Tables

**Table 1:** Effect of Leu and Val on meat quality of finishing pigs.

Items	Con	Leu	Val	SEM	*p*-Value
pH_45min_	6.23	6.52	6.56	0.08	0.17
pH_24h_	5.68	5.60	5.58	0.02	0.09
L*_45min_	43.53	43.56	43.28	0.37	0.94
L*_24h_	51.31	52.17	52.70	0.89	0.83
a*_45min_	15.31	16.40	15.23	0.36	0.35
a*_24h_	13.65	14.05	14.76	0.48	0.65
b*_45min_	4.45	4.58	4.26	0.18	0.79
b*_24h_	8.69	9.20	10.22	0.53	0.52
Drip loss, %	2.39	2.86	3.16	0.23	0.39
Cook loss, %	36.23	33.35	32.14	1.64	0.61
Shear force, N	45.41 ^a^	36.21 ^b^	37.97 ^b^	1.14	<0.001
IMF, %	3.70 ^a^	3.00 ^b^	2.89 ^b^	0.10	0.009

Data are presented as mean and SEM. Mean values with different letters were considered significantly different. Con: basal diet supplemented with alanine isonitrogenous with the Leu group; Leu: basal diet supplemented with 1% Leu; Val: basal diet supplemented with Val isonitrogenous with the Leu group; IMF: Intramuscular fat; SEM, standard error of mean.

**Table 2:** Effect of Leu and Val supplementation on fatty acid composition in LL muscle of finishing pigs (% of total fatty acids).

Items	Con	Leu	Val	SEM	*p*-Value
C12:0	0.22	0.21	0.22	0.001	0.152
C14:0	1.65 ^a^	1.55 ^b^	1.45 ^c^	0.022	<0.001
C16:0	31.52	30.73	30.68	0.168	0.065
C18:0	17.61 ^a^	16.83 ^b^	16.38 ^b^	0.148	<0.001
C20:0	1.26 ^a^	1.15 ^b^	1.24 ^a^	0.014	<0.001
C22:0	0.62 ^a^	0.30 ^c^	0.43 ^b^	0.033	<0.001
C16:1	4.24 ^a^	4.04 ^b^	4.09 ^ab^	0.035	0.035
C18:1	29.80 ^c^	32.03 ^b^	33.11 ^a^	0.353	<0.001
C22:1	1.61	1.57	1.55	0.01	0.147
C18:2	8.32 ^ab^	8.41 ^a^	7.71 ^b^	0.121	0.025
C18:3 n6	0.28	0.26	0.26	0.004	0.001
C18:3 n3	0.28	0.29	0.27	0.002	0.556
C20:3 n6	0.32 ^a^	0.27 ^b^	0.31 ^a^	0.006	<0.001
C20:3 n3	0.31 ^b^	0.37 ^a^	0.32 ^b^	0.007	<0.001
C20:4 n6	1.44	1.38	1.41	0.012	<0.001
C20:5 n3(EPA)	0.30 ^b^	0.33 ^a^	0.31 ^b^	0.005	0.008
C22:6 n3(DHA)	0.24 ^c^	0.29 ^a^	0.27 ^b^	0.006	<0.001
n-3 PUFA	1.12 ^c^	1.28 ^a^	1.16 ^b^	0.017	<0.001
n-6 PUFA	10.36 ^a^	10.31 ^ab^	9.69 ^b^	0.119	0.026
n-6/n-3 PUFA	9.23 ^a^	8.08 ^b^	8.32 ^b^	0.149	<0.001

Data are presented as mean and SEM. Mean values with different letters were considered significantly different. EPA, eicosapentaenoic acid; DHA, docosahexaenoic acid; Con: basal diet supplemented with alanine isonitrogenous with the Leu group; Leu: basal diet supplemented with 1% Leu; Val: basal diet supplemented with Val isonitrogenous with the Leu group; SEM, standard error of mean.

**Table 3:** Effect of Leu and Val supplementation on antioxidant enzyme activities of finishing pigs.

Items	Con	Leu	Val	SEM	*p*-Value
Serum					
MDA, nmol/mL	3.63	3.55	3.68	0.086	0.846
T-AOC, U/mL	0.77	0.82	0.80	0.011	0.190
T-SOD, U/mL	55.72 ^b^	74.91 ^a^	70.92 ^a^	2.129	<0.001
GSH-Px, U/mL	1131.83 ^b^	1240.70 ^a^	1189.57 ^ab^	17.364	0.026
CAT, U/mL	4.00 ^ab^	4.51 ^a^	3.84 ^b^	0.11	0.030
Liver					
MDA, nmol/mg protein	7.94 ^a^	4.93 ^c^	6.74 ^b^	0.346	<0.001
T-AOC, U/mg protein	0.36 ^b^	0.44 ^a^	0.39 ^ab^	0.011	0.003
T-SOD, U/mg protein	32.91 ^b^	39.57 ^a^	38.74 ^a^	0.933	0.001
GSH-Px, U/mg protein	74.26 ^b^	82.54 ^a^	79.91 ^a^	0.961	<0.001
CAT, U/mg protein	20.50	21.96	21.26	0.284	0.105
LL Muscle					
MDA, nmol/mg protein	8.99 ^a^	5.26 ^c^	6.38 ^b^	0.42	<0.001
T-AOC, U/mg protein	0.12 ^c^	0.54 ^a^	0.38 ^b^	0.04	<0.001
T-SOD, U/mg protein	155.18 ^c^	214.88 ^a^	191.98 ^b^	6.71	<0.001
GSH-Px, U/mg protein	101.66 ^b^	209.24 ^a^	223.94 ^a^	13.52	<0.001
CAT, U/mg protein	2.60 ^b^	4.64 ^a^	3.05 ^b^	0.31	0.01

Data are presented as mean and SEM. Mean values with different letters were considered significantly different. MDA, malondialdehyde; T-AOC, total antioxidant capacity; T-SOD, total superoxide dismutase; GSH-Px, glutathione peroxidase; CAT, catalase. Con: basal diet supplemented with alanine isonitrogenous with the Leu group; Leu: basal diet supplemented with 1% Leu; Val: basal diet supplemented with Val isonitrogenous with the Leu group; SEM, standard error of mean.

## Data Availability

The raw datasets used and analyzed during the current study are available from the corresponding author upon reasonable request.

## Supplementary Materials

The following supporting information can be downloaded at: https://www.mdpi.com/article/10.3390/ani16152317/s1, Table S1: Ingredient composition and nutritional levels of the diet; Table S2: RT-PCR primer sequences. Figure S1. Correlation network among shear force, myosin heavy chain, and antioxidant-related genes in meat of finishing pigs. Finishing pigs were fed either a basal diet or basal diets supplemented with Leu or Val for 30 days (*n* = 6). Figure S2. Correlation network between lipid metabolism genes and antioxidant-related genes in meat of finishing pigs. Finishing pigs were fed either a basal diet or basal diets supplemented with Leu or Val for 30 days (*n* = 6).

## Abbreviations

The following abbreviations are used in this manuscript:

BCAAs	Branched-Chain Amino Acids
Leu	Leucine
Val	Valine
LL	Longissimus Lumborum
CSA	Cross-Sectional Area
SFA	Saturated Fatty Acid
MUFA	Monounsaturated Fatty Acid
PUFA	Polyunsaturated Fatty Acid
T-SOD	Total Superoxide Dismutase
T-AOC	Total Antioxidant Capacity
GSH-Px	Glutathione Peroxidase
CAT	Catalase
MDA	Malondialdehyde
IMP	Inosine monophosphate
