# Supplementary material for: Leucine and Valine Improve Pork Tenderness by Driving Myofiber Transition, Optimizing Lipid Profiles and Enhancing Antioxidant Defense
Source: Animals (Basel). 2026 Jul 28;16(15):2317. doi: 10.3390/ani16152317 (PMC13463578; doi:10.3390/ani16152317)
Supplement: Supplementary file 1 [file animals-16-02317-s001.zip › animals-4426116-supplementary.pdf]

Table S1 Ingredient composition and nutritional levels of the diet

Diet ingredients（%）	Con	Leu	Val
Corn	70.58	70.30	70.37
Soybean meal (46.2% CP)	10.93	10.89	10.90
Rice bran meal	7.95	7.92	7.93
Wheat bran	5.96	5.91	5.94
Soybean oil	0.99	0.99	0.99
L-Lysine (98%)	0.30	0.30	0.30
Dicalcium phosphate	0.40	0.40	0.40
Limestone	0.89	0.89	0.89
Premix[a]	1.00	1.00	1.00
Salt	0.40	0.40	0.40
Alanine	0.60	0.00	0.00
Leucine	0.00	1.00	0.00
Valine	0.00	0.00	0.88
Total	100.00	100.00	100.00
Nutrient levels（%）			
ME[bc] (Mcal/kg)	3.04	3.03	3.03
Crude protein	13.42	13.21	13.64
Lysine[b]	0.58	0.58	0.58
Methionine[b]	0.23	0.23	0.23
Threonine[b]	0.48	0.48	0.48
Tryptophan[b]	0.15	0.15	0.15
Calcium	0.48	0.48	0.49
Total phosphorus	0.61	0.62	0.62
Available phosphorus[b]	0.14	0.14	0.14

[a] Supplied per kilogram of diet: 4200 IU vitamin A, 1600 IU vitamin D3, 20 IU vitamin E, 1.0 mg vitamin K, 20 mg niacin, 11.2 mg pantothenic acid, 12 μg vitamin B12, 0.1 mg biotin, 0.4 mg folic acid, 2 mg thiamin, 4 mg riboflavin, 80 mg Zn, 20 mg Mn, 80 mg Fe, 11 mg Cu, 0.2 mg I, and 0.2 mg Se.

[b] calculated values.

[c] ME: Metabolizable Energy.

Table S2 RT-PCR primer sequences

Gene	Gene Bank ID	Forward Sequence (5'-3')	Amplicon length (bp)	Efficiency (%)
β-actin	XM_021086047.1	F: AATCCTGCGGCATCCACGAAAC<br>R: GTGTTGGCGTAGAGGTCCTTGC	82	98.2
Sod1	NM_001190422.1	F: CTCTCGGGAGACCATTCCATCATTG<br>R: TTCTTCATTTCCACCTCTGCCCAAG	84	97.8
Cat	NM_214301.2	F: CGCCTATTTGCCTATCCTGACACTC<br>R: GCACGGAAGGGACAGTTCACAG	83	99.4
GPx	NM_214201.1	F: CACGCTCGGTGTATGCCTTCTC<br>R: GCAGCTCATTCATCTGGGTGTAGTC	150	98.7
Gst	NM_213850.2	F: CATCGCCACCAAGTACAACCTCTAC<br>R: TTCACCCAAATCTGCCACACCTTC	91	97.9
Nrf2	XM_021075133.1	F: AGCACAACACATCCCGTCAGAAAC<br>R: GAGCCTGGTTAGGAGCAATGAAGAC	134	99.4
Acc	XM_021066238.1	F: AATCAAAGAGGTTCCAGGCACAGTC<br>R: CGTCAGCATGTCAGAAGGCAGAG	149	99.2
Fas	NM_001099930.1	F: TGGGCATGGTGAACTGTCTC<br>R: GCGTGGTTGTTGGAAAGGTC	976	98.3
Ampk	NM_001167633.1	F: GCAACAAGCCCACCTGATTCTTTTC<br>R: GTATGGCGTGCCCTTGGAGTTTC	104	98.4
Sirt1	NM_001145750.2	F: CGGCAGGAGAAGGAAACAATGGG<br>R: TCGTCGTCGTCGTCGTAGAAGTC	88	97.7
Pgc-1α	NM_213963.2	F: GGCAGAGGCAGATGTGTTCTTCC<br>R: CGGCTCGGATTTCCTGGTCTTG	126	98.1
Fabp3	NM_001099931.1	F: TTGTGACACTGGATGGAGGC<br>R: TAAGTGCGAGTGCAAACTGC	136	97.5
Fabp4	NM_001002817.1	F: TGAAAGAAGTGGGAGTGGGC<br>R: CTGGCCCAATTTGAAGGCAA	146	98.4
MyHC Ⅰ	NM_213855.2	F: GCTCCGTCTTCTTTCCTTGC<br>R: GTCCTCCTTCACAGTCACCG	312	99.1
MyHC Ⅱa	XM_021066217.1	F: CACTGCCTTTATCCACCAGC<br>R: GCAGGACGAGTCAGCTTCAT	267	98.4
MyHC Ⅱb	NM_001123141.1	F: GGTACATCTAGTGCCCTGCT<br>R: AGTTGTCGTTCCTCACGGTC	770	98.3
MyHC Ⅱx	NM_001104951.2	F: CTTTCCTCTTCACTGGCGCA<br>R: GATTCTGCTTGGGAAGCCCT	290	98.5

Abbreviations: *Sod1*, superoxide dismutase 1; *Cat*, catalase; *GPx*, glutathione peroxidase; *Gst*, glutathione s-transferase; *Nrf2*, nuclear factor erythroid 2-related factor 2; *Acc*, acetyl CoA carboxylase; *Fas*, fatty acid synthase; *Ampk*, adenosine monophosphate-activated protein kinase; *Sirt1*, sirtuin 1; *Pgc-1α*, peroxisome proliferator activated receptor-γ coactivator-1 alpha; *Fabp*, fatty acid-binding protein; *MyHC*, Myosin heavy chain.

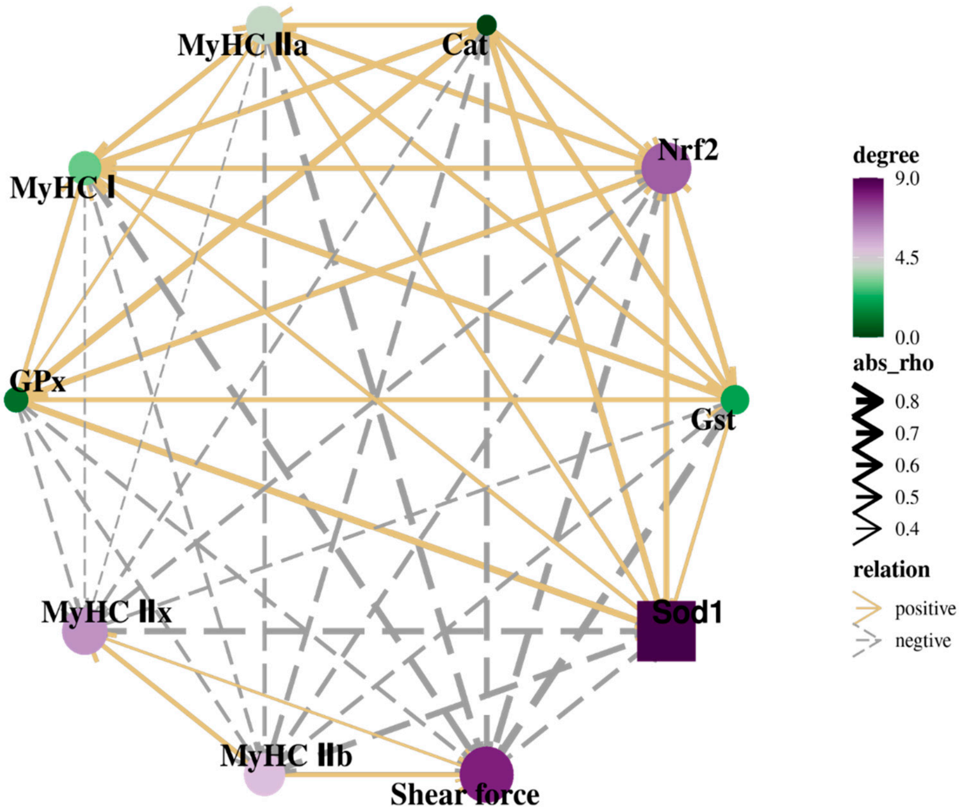

Figure S1. Correlation network among shear force, myosin heavy chain and antioxidant-related genes in meat of finishing pigs. Finishing pigs were fed either a basal diet or basal diets supplemented with Leu or Val for 30 days (n = 6).

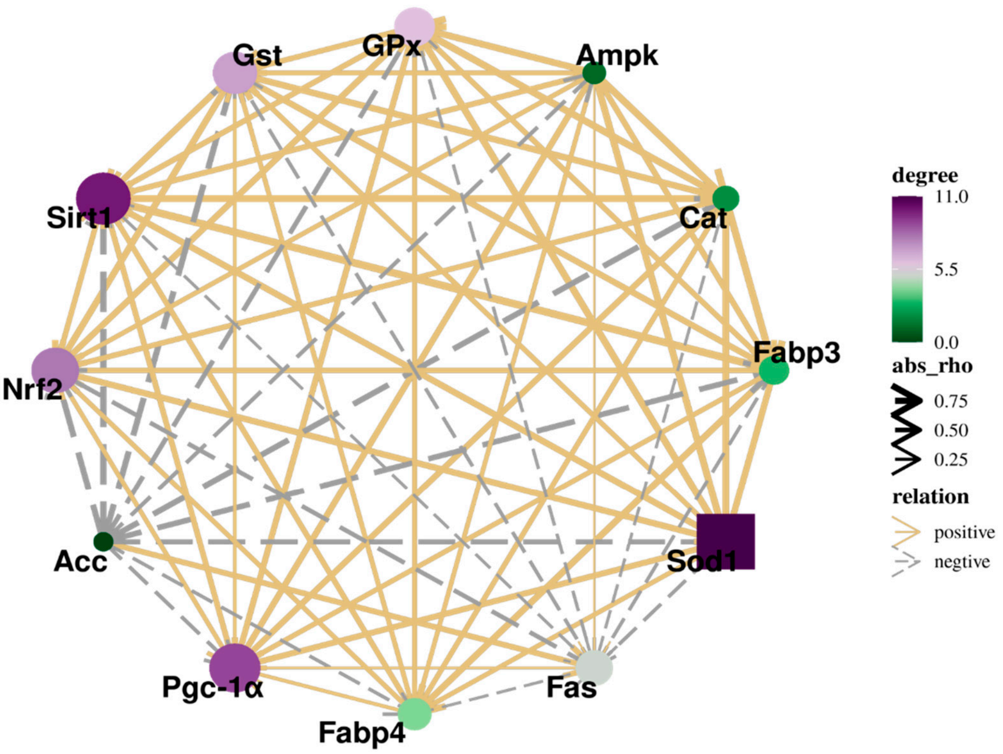

Figure S2. Correlation network between lipid metabolism genes and antioxidant-related genes in meat of finishing pigs. Finishing pigs were fed either a basal diet or basal diets supplemented with Leu or Val for 30 days (n = 6).
